# Recognizing Haemophagocytic Lymphohistiocytosis in an HIV Patient With Disseminated Tuberculosis: Not Every Fever is Sepsis

**DOI:** 10.7759/cureus.94772

**Published:** 2025-10-17

**Authors:** Kawser Ahmed, Ashfaq Hamid

**Affiliations:** 1 Acute Internal Medicine, Northampton General Hospital NHS Trust, Northampton, GBR

**Keywords:** acute care medicine, haemophagocytic lymphohistiocytosis (hlh), hiv tuberculosis, hscore, hyperinflammation

## Abstract

Haemophagocytic lymphohistiocytosis (HLH) is a rare but potentially life-threatening condition that causes hyperinflammation, extensive tissue destruction, multi-organ failure (MOF) and death due to uncontrolled activation and proliferation of T cells and macrophages. HLH can be primary (genetic) and secondary (acquired). Primary HLH occurs due to genetic mutations, and secondary HLH is often triggered by autoimmune diseases, infections (commonly viral, bacterial and parasites) and malignancies, such as lymphoma. We report a case of secondary HLH in a 43-year-old man with an untreated human immunodeficiency virus (HIV) infection and newly diagnosed disseminated tuberculosis who was admitted to the acute medicine ward with some non-specific gastrointestinal symptoms. Nevertheless, he was treated with guideline-directed antibiotics, but his condition failed to improve with persistent high-grade fever, haemodynamic instability, splenomegaly, pancytopenia, transaminitis and hyperferritinaemia (ferritin: >18,000 μg/L). Hence, the multidisciplinary team (MDT) initiated a workup including the Haemophagocytic Lymphohistiocytosis Diagnostic Score (HScore) and bone marrow biopsy, along with viral screening, tuberculosis (TB) tests and computed tomography (CT) of the chest, abdomen and pelvis, followed by bronchoscopy. The clinical diagnosis of HLH was established based on the highly supportive clinical and laboratory criteria, as reflected by an extremely high HScore (272), despite the absence of haemophagocytosis on bone marrow biopsy. Later, the bronchial washing confirmed the detection of *Mycobacterium tuberculosis*. Our rheumatology, haematology and acute medicine team consensually agreed to start intravenous (IV) pulse methylprednisolone for three days, followed by antiretroviral and anti-tubercular regimens. This case underscores the diagnostic challenge of HLH in untreated patients with HIV and disseminated TB, where the clinical manifestations can resemble sepsis, cytokine storm or systemic inflammatory response syndrome (SIRS). Ultimately, early identification and timely intervention with immunosuppressive therapy are crucial for the favourable outcome of patients with HLH.

## Introduction

Haemophagocytic lymphohistiocytosis (HLH) is a systemic hyperinflammatory disorder caused by a dysregulated immune system, which leads to cytokine storm, widespread tissue injury and multi-organ failure, and is associated with a significantly high mortality rate (ranging from 26.5% to 748% in reported series) if left untreated [1]. Primary HLH is more common in children, and the defective genes are *UNC13D*, *PRF1*, *STX11* and *STXBP2* [2]. Secondary HLH is primarily found in adults and is typically associated with external triggers such as autoimmune disorders, infections, cancers, immunodeficiency states and medications [3]. Regardless of the aetiology, the pathophysiology culminates in a 'cytokine storm' due to the uncontrolled activation and proliferation of cytotoxic T cells and macrophages [4]. The diagnosis of HLH, especially in the acute medicine ward, is truly challenging due to its similar presentation to severe sepsis, systemic inflammatory response syndrome (SIRS) or cytokine storm [5]. The typical presentation of HLH is persistent high fever, lymphadenopathy, organomegaly, cytopaenias, hyperferritinaemia and transaminitis [6]. The hallmark of HLH is the presence of haemophagocytes in the bone marrow; however, this finding is time-consuming to detect and may be negative in the early stages of the disease process [7]. Therefore, diagnostic tools such as the HLH-2004 criteria, as well as the Haemophagocytic Lymphohistiocytosis Diagnostic Score (HScore), are the initial assessment methods to pave the diagnostic pathway [8].

Secondary HLH, especially infections triggered, is particularly important for immunosuppressed patients. HLH due to human immunodeficiency virus (HIV) is commonly associated with opportunistic infections such as tuberculosis (TB), Epstein-Barr virus (EBV), cytomegalovirus (CMV) and histoplasmosis [9]. Disseminated TB is a recognized trigger for secondary HLH, particularly in advanced HIV infection characterized by low CD4 counts and high viral loads [10]. It should be noted that immune reconstitution inflammatory syndrome (IRIS) is usually diagnosed after the initiation of antiretroviral therapy (ART), whereas the patient in this case report had untreated HIV [11]. Therefore, this case report highlights the importance of early recognition and treatment of HLH in immunocompromised patients presenting with systemic inflammatory features unresponsive to standard therapy. The treatment approach of primary HLH, which often involves allogenic bone marrow transplantation combined with steroids and chemotherapy, differs significantly from the management of secondary HLH [12].

## Case presentation

A 43-year-old male patient presented to the emergency department with multiple episodes of diarrhoea, abdominal pain and weight loss for one week in conjunction with malaise. He denied any associated vomiting, haematemesis, PR bleeding, faecal urgency, headache, chest pain or short of breath (SOB).

His past medical illness was significant for untreated HIV, diagnosed in 2023, and pulmonary embolism in 2024. He was not on any regular medications and had no known allergies.

On initial examinations, his National Early Warning Score (NEWS) was 2. Observations revealed tachycardia (heart rate (HR): 112 bpm, regular), blood pressure of 120/86 mmHg, temperature of >36.1°C, respiratory rate (RR) of 18 breaths/minute and saturations of 96% on room air. He opened his bowel six times with type 5 stool as per the Bristol stool chart over the past 24 hours. The patient was alert and did not require any supplemental oxygen therapy. Abdominal examinations showed a soft but tender abdomen, which limited further evaluation. Additionally, there were a few small, non-tender anterior cervical lymphadenopathies. His other systemic reviews were unremarkable. Initial venous blood gas (VBG) showed pH of 7.8, haemoglobin (Hb) of 78 g/L, hypokalaemia (potassium (K^+^): 2.9 mmol/L), hyponatraemia (sodium (Na^+^): 129 mmol/L) and lactate of 1.3 mmol/L. However, the routine bloods showed the following findings (Table 1), where some discrepancies were noted due to differences in values between the VBG machine and laboratory values.

**Table 1:** Laboratory analysis Hb: haemoglobin, Hct: haematocrit, WCC: white cell count, Plt: platelet count, CRP: C-reactive protein, Na: sodium, K: potassium, eGFR: estimated glomerular filtration rate, ALP: alkaline phosphatase, ALT: alanine aminotransferase, APTT: activated partial thromboplastin time, INR: international normalized ratio, TSAT: transferrin saturation, TIBC: total iron-binding capacity, LDH: lactate dehydrogenase, CXR: chest X-ray, cCa²⁺: corrected calcium

Test	Result	Normal range
Hb	71 g/L	120-150 g/L
Hct	23%	40%-50%
WCC	7.3 × 10⁹/L	4-10 × 10⁹/L
Neutrophil	6.87 × 10⁹/L	1.5-6.5 × 10⁹/L
Plt	139 × 10⁹/L	150-400 × 10⁹/L
CRP	275 mg/L	<5 mg/L
Na	130 mmol/L	135-145 mmol/L
K	3.1 mmol/L	3.5-5.1 mmol/L
Urea	4.5 mg/dL	7-20 mg/dL
Creatinine	65 mg/dL	59-101 mg/dL
eGFR	>90 mL/min	>60 mL/min
Total protein	64 g/L	63-82 g/L
Albumin	29 g/L	35-50 g/L
cCa²⁺	2.32 mmol/L	2.25-2.65 mmol/L
Bilirubin	17 μmol/L	3-22 μmol/L
ALP	66 IU/L	38-126 IU/L
ALT	94 IU/L	9-52 IU/L
APTT	36 seconds	22-30 seconds
INR	1.1	0.8-1.2
TSAT	8%	20%-50%
Iron	2 μmol/L	14-32 μmol/L
TIBC	26 μmol/L	44-71 μmol/L
LDH	725 IU/L	13-225 IU/L
CXR	Unremarkable	-

Based on his presentation, infectious diarrhoea was suspected initially, and he was treated with intravenous (IV) empiric antibiotic and IV fluid, and faecal testings were requested. However, stool cultures, *Clostridium difficile* toxin (CDT) and CMV PCR returned negative. Infectious mononucleosis was also excluded with a negative Monospot test.

His initial differentials were acute diarrhoea, probably due to infectious causes, such as CMV, HIV wasting syndrome and infectious mononucleosis. His stool test was negative, along with a negative Monospot test. However, he was initially managed with IV antibiotics, IV fluids and maintaining stool chart.

**Figure 1:**
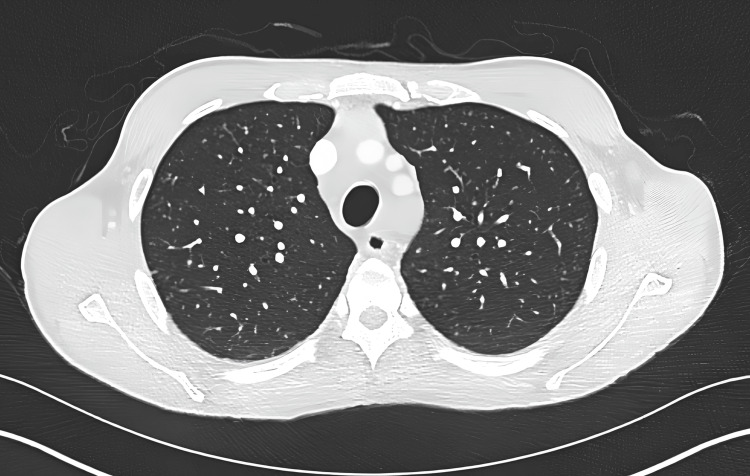
CT of the thorax (axial view) showing multiple tiny lung nodules bilaterally with the differential of calcification and miliary TB CT: computed tomography, TB: tuberculosis

**Figure 2:**
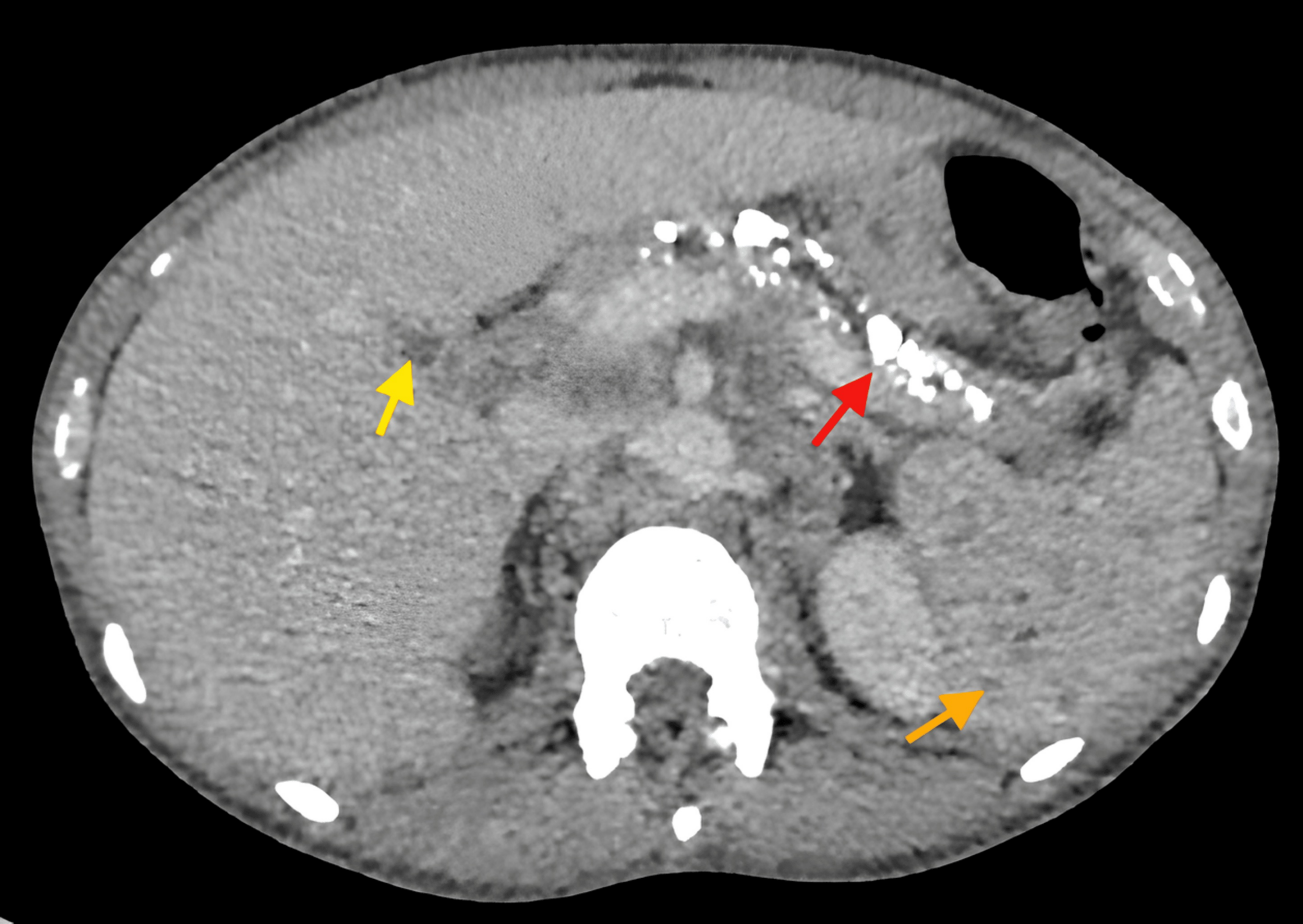
CT of the abdomen (axial view) showing multiple tiny hypoenhancing splenic nodules, extensive pancreatic calcification and one hypoenhancing liver nodule Golden arrow: spleen, red arrow: pancreas, yellow arrow: liver CT: computed tomography

Despite the supportive treatment as per Trust protocol, his condition deteriorated over the following days. He was experiencing persistently high-grade fever (>39.8°C), violent rigors confused as seizure-like activities, new-onset confusion and progressive breathlessness. His NEWS remained persistently high. Therefore, a computed tomography (CT) of the chest, abdomen and pelvis was performed, where positive findings are noted in Figure 1 and Figure 2.

Serological testing is positive for both anti-HIV-1 and p24, with a high viral load (1.70 × 106 copies/mL). Epstein-Barr virus (EBV) and parvovirus B19 serology were negative. CMV PCR and *C. difficile* PCR remained undetectable. However, bronchoscopy followed by bronchial washing confirmed *Mycobacterium tuberculosis* (TB).

Despite broad-spectrum antibiotic, antiviral and anti-TB regimens, his clinical conditions deteriorated by day 10 of admission (Table 2). He exhibited continuous high-grade fever (40.6°C), tachycardia (HR: 128 bpm, regular), low pulse pressure, hypotension and tachypnoea (RR: 32 breaths/minute), and required supplemental oxygen. He had a pseudoseizure due to violent rigor. Repeat examinations revealed cervical lymphadenopathy and splenomegaly. He underwent peri-arrest call, and repeat CT and CXR revealed no new changes. At this point, HLH was clinically suspected due to the constellation of features that are characteristic of this syndrome. These findings prompted involvement of a multidisciplinary team (MDT) comprising acute medicine, rheumatology, haematology and infectious disease specialists. The MDT outcome was to follow the British Society of Rheumatology HLH pathway, and the calculated Haemophagocytic Lymphohistiocytosis Diagnostic Score (HScore) is shown in Table 3.

**Table 2:** Laboratory results of day 10. Hb: haemoglobin, Hct: haematocrit, WCC: white cell count, Plt: platelet count, CRP: C-reactive protein, Na⁺: sodium, K⁺: potassium, eGFR: estimated glomerular filtration rate, ALP: alkaline phosphatase, ALT: alanine aminotransferase, AST: aspartate aminotransferase, APTT: activated partial thromboplastin time, INR: international normalized ratio, LDH: lactate dehydrogenase, cCa²⁺: corrected calcium

Test	Result	Normal range
Hb	65 g/L	120-150 g/L
Hct	22%	40%-50%
WCC	1.1 × 10⁹/L	4-10 × 10⁹/L
Neutrophil	0.97 × 10⁹/L	1.5-6.5 × 10⁹/L
Plt	61 × 10⁹/L	150-400 × 10⁹/L
CRP	326 mg/L	<5 mg/L
Na⁺	134 mmol/L	135-145 mmol/L
K⁺	3.2 mmol/L	3.5-5.1 mmol/L
Urea	3.7 mg/dL	7-20 mg/dL
Creatinine	52 mg/dL	59-101 mg/dL
eGFR	>90 mL/min	>60 mL/min
Ferritin	18,283 μg/L	30-400 μg/L
Total protein	53 g/L	63-82 g/L
Albumin	21 g/L	35-50 g/L
cCa²⁺	2.32 mmol/L	2.25-2.65 mmol/L
Total serum bilirubin	14 μmol/L	3-22 μmol/L
ALP	145 IU/L	38-126 IU/L
ALT	66 IU/L	9-52 IU/L
AST	222 IU/L	5-40 IU/L
Triglycerides	2.7 mmol/L	0.0-2.3 mmol/L
APTT	41 seconds	22-30 seconds
INR	1.2	0.8-1.2
Fibrinogen	5.1 g/L	1.8-5.0 g/L
LDH	1,131 IU/L	13-225 IU/L

**Table 3:** HScore HScore: Haemophagocytic Lymphohistiocytosis Diagnostic Score, Temp: temperature (°C), cytopaenia: reduction in blood cell lineages, AST: aspartate aminotransferase (IU/L), BN: bone marrow aspirate/biopsy, HIV: human immunodeficiency virus

Traits	Positive features	Score
Known immunosuppression	Yes (HIV)	18
Temperature	>39.4°C	49
Organomegaly	Yes (splenomegaly)	23
Cytopaenia	3 lineages	34
Ferritin	>6,000 μg/L	50
Triglyceride	2.7 mmol/L	44
Fibrinogen	>250 mg/dL	0
AST	>30 IU/L	19
Haemophagocytosis on BN	No	0

The calculated HScore was 272, consistent with a high probability of HLH. The MDT mutually agreed to label it as HLH in the context of untreated HIV infection and disseminated TB. Therefore, he was promptly treated with IV pulse methyl prednisolone 1 gm for three consecutive days along with antiretroviral and anti-TB regimen and supportive care. After three days of pulse methylprednisolone, his condition improved both clinically and biochemically, which also supported the diagnosis of HLH. The next day, he was transferred to a tertiary HLH centre for further management and was followed up there.

## Discussion

Haemophagocytic lymphohistiocytosis is a severe, life-threatening hyperinflammatory syndrome caused by excessive immune activation, most commonly involving T lymphocytes and macrophages [13]. This immune dysregulation results in an uncontrolled cytokine storm, leading to multiorgan failure and death if not recognized and treated early [14]. HLH is broadly classified into primary (genetic) and secondary (acquired) types [15]. While primary HLH typically manifests in children due to mutations in genes such as *PRF1*, *UNC13D*, *STX11* and *STXBP2*, secondary HLH is more common in adults and is usually triggered by infections, cancers, autoimmune disorders or immunosuppressive therapy [16].

We report a case of a 43-year-old man with untreated HIV infection and disseminated tuberculosis (TB) presenting with features highly suggestive of HLH. The constellation of signs and laboratory findings, including persistent high fever, trilineage cytopaenias, splenomegaly, marked hyperferritinaemia, hypertriglyceridaemia and transaminitis, along with an exceptionally high HScore of 272, strongly suggested the diagnosis of HLH. Subsequently, the MDT confirmed the diagnosis based on the current diagnostic guideline, although the bone marrow was negative for haemophagocytosis.

HLH in HIV-positive patients poses a significant diagnostic challenge due to overlapping features with opportunistic infections and immune reconstitution inflammatory syndrome (IRIS) [9]. Although HIV does not directly cause HLH, the opportunistic infections associated with HIV, such as EBV, CMV, histoplasmosis and TB, are known to cause secondary/acquired HLH [10]. In our patient, TB was the inciting factor on the background of HIV, as we excluded the other opportunistic infections based on the blood test, history and examinations.

TB-associated HLH is becoming more widely acknowledged, especially in immunocompromised individuals, in the literature. A recent systematic review highlighted that TB is one of the most prevalent infectious causes of HLH in HIV-positive patients [17]. As seen in our patient, disseminated TB is frequently linked to HLH and manifests as fever, weight loss, cytopaenias and raised inflammatory markers.

Distinguishing HLH from severe sepsis or systemic inflammatory response syndrome (SIRS) is a major clinical challenge [18]. Since our patient was not on antiretroviral therapy (ART) when symptoms began, immune reconstitution inflammatory syndrome (IRIS) was excluded as an initial diagnosis. Furthermore, the presence of extreme hyperferritinaemia (18,000ug/L in this case), in conjunction with progressive cytopaenias and hepatic dysfunction, strongly favoured HLH over uncomplicated sepsis or disseminated TB. Consideration of HLH is prudent by these clinical and biochemical indicators when the patient was not improving with the conventional antimicrobial treatment.

The HLH-2004 diagnostic criteria or the more recent revised diagnostic guidelines are used to diagnose HLH. These criteria need a molecular diagnosis or the fulfillment of more than five out of eight clinical and laboratory parameters [19]. Our patient satisfied the requirements of fever, splenomegaly, cytopaenias in three lineages, hyperferritinaemia and hypertriglyceridaemia. Despite being the hallmark of the bone marrow or tissue biopsies, haemophagocytosis is neither sensitive nor specific, although it might be absent in the early stage of the disease [20]. Therefore, physicians should not exclusively rely on the biopsy result, as without the immediate treatment, patients might deteriorate quickly [21]. Hence, the HScore has been established with the combinations of clinical and laboratory markers to determine the probability of adult HLH. A score more than 250 is associated with a >99% chance of adult HLH [22]. Our patient's HScore of 272 provided strong support for the diagnosis of HLH, facilitating the urgent therapeutic action.

Two main strategies are needed to manage secondary HLH: immunosuppression to control hyperinflammation and specific treatment of the secondary causes [23]. The equilibrium is more delicate in infection-triggered secondary HLH. The mainstay of treatment of HLH is high-dose steroids [24]. Our patient had a dramatic improvement in both clinical and biochemical markers after starting the intravenous pulse methylprednisolone (1 gm daily for three subsequent days). At the same time, an anti-TB regimen was started. The requirement of high-dose steroids in the middle of an active infection makes the management of TB in HLH more challenging [25]. According to recent research, steroids and anti-TB regimens work better together to increase the survival rates in TB-associated HLH [26]. Antiretroviral therapy was also introduced, although its timing was carefully considered to reduce the risk of IRIS.

Etoposide, an anti-cancer drug, has an established role in paediatric HLH and in severe or refractory adult HLH [27]. However, our patient did not receive this treatment. Referral to a tertiary HLH centre, along with an MDT approach, is key to managing HLH, which was also contemplated in our patient [21]. The complexities of HLH treatment demand such collaboration, which is crucial.

Recent research highlights the increasing prevalence of HLH among immunocompromised individuals, especially those with co-infection of HIV and TB [17]. A recent review article stressed that HLH should be considered in patients who presented with fever, cytopaenia and raised ferritin, even if the alternative diagnosis of sepsis and tuberculosis is suspected initially [28]. Data from the HLH registry suggests that the outcome is fatal if the diagnosis and treatment are delayed [29]. Therefore, it is crucial to use the diagnostic algorithm rather than waiting for a bone marrow biopsy to start immunosuppressant and the treatment of inciting factors [30].

## Conclusions

This case underscores the critical importance of maintaining a high index of suspicion for secondary HLH in immunocompromised patients, specifically those with co-existing untreated HIV and disseminated TB, who present with persistent systemic inflammation and multi-organ dysfunction. Further research is warranted to develop simplified diagnostic pathways for HLH in low-resource settings. This case reinforces the need for institutional protocols to streamline referral to specialized centres. Greater awareness and training on HLH recognition among acute care teams may facilitate earlier intervention and improve survival.
